# Effects of Severe Acute Respiratory Syndrome Coronavirus Vaccination on Reinfection: A Community-Based Retrospective Cohort Study

**DOI:** 10.3390/vaccines11091408

**Published:** 2023-08-23

**Authors:** Hyerin Gim, Seul Lee, Haesook Seo, Yumi Park, Byung Chul Chun

**Affiliations:** 1 Infectious Disease Research Center, Citizens’ Health Bureau, Seoul Metropolitan Government, Seoul 04524, Republic of Korea; linflower@seoul.go.kr (H.G.); seul0710@gmail.com (S.L.); 27723@seoul.go.kr (H.S.); 2 Department of Epidemiology & Health Informatics, Graduate School of Public Health, Korea University, Seoul 02841, Republic of Korea; 3 Citizens’ Health Bureau, Seoul Metropolitan Government, Seoul 04524, Republic of Korea; soandho1@hanmail.net; 4 Department of Preventive Medicine, Korea University College of Medicine, Seoul 02841, Republic of Korea

**Keywords:** COVID-19, epidemiology, reinfection, vaccination, symptoms

## Abstract

Background: Coronavirus disease 2019 (COVID-19) is a disease that is characterized by frequent reinfection. However, the factors influencing reinfection remain poorly elucidated, particularly regarding the effect of COVID-19 vaccination on preventing reinfection and its effects on symptomatology and the interval until reinfection. Methods: This retrospective cohort study examined patients with severe acute respiratory syndrome coronavirus reinfection between January 2020 and February 2022. This study included patients aged >17 years who were reinfected at least 90 days between two infections with severe acute respiratory syndrome coronavirus. The main outcome measure was a reduction in symptoms during reinfection, and reinfection interval. Results: Overall, 712 patients (average age: 40.52 ± 16.41 years; 312 males) were included. The reduction rate of symptoms at reinfection than that at first infection was significantly higher in the vaccinated group than in the unvaccinated group (*p* < 0.001). The average reinfection interval was 265.81 days. The interval between the first and second infection was 63.47 days longer in the vaccinated group than in the unvaccinated group. The interval was also 57.23 days, significantly longer in the asymptomatic group than in the symptomatic group (*p* < 0.001). Conclusions: Besides its role in preventing severe acute respiratory syndrome coronavirus infection, vaccination reduces the rate of symptomatic reinfection and increases the reinfection interval; thus, it is necessary to be vaccinated even after a previous infection. The findings may inform the decision to avail COVID-19 vaccination.

## 1. Introduction

Patients vaccinated against coronavirus disease 2019 (COVID-19) generally have a significantly lower risk of recurrent infection, symptomatic disease, and severity [1,2,3,4]. However, studies comparing clinical symptoms and severity in the same person are scarce. Further, for reinfection case confirmation, real-time polymerase chain reaction (RT-PCR) testing was only performed for the symptomatic cases in previous studies [3,5], and among COVID-19 cases, 40.5% were asymptomatic [6]. A bias in the studies of asymptomatic cases is likely. With the Omicron variant’s emergence, which is known to have a high percentage of asymptomatic infections compared with the other variants [7,8,9], the proportion of completely asymptomatic cases may be much larger. Therefore, it is necessary to study populations of asymptomatic and symptomatic cases in a community-based retrospective cohort.

Severe acute respiratory syndrome coronavirus (SARS-CoV-2) infection or vaccination suppresses reinfection by increasing neutralizing antibodies [10,11]. In previous studies, natural immunity was shown to lower the risk of reinfection [12,13] and 20-month hospitalization [14]. Natural Killer cells (NK cells) play a major role in preventing viral infections and eliminating tumors, as well as for the work of the immune system [15]. Natural immunity such as NK cell and vaccination immunity reduce reinfection [14] and lower the risk of additional 9-month hospitalization [12]. The COVID-19 reinfection interval, which is the interval between consecutive infections, is the period from the first infection date to reinfection and represents the body’s immune protection period against reinfection [10,16]. The reinfection interval after the first SARS-CoV-2 infection was found to be 6–8 months [17]. A protection period of more than 1 year is achieved when vaccination is performed despite SARS-CoV-2 infection [18]. During the Omicron variant spread, protective immunity against COVID-19 was reduced to 28.6–35.6% 10 weeks after vaccination [19]. As the COVID-19 pandemic continues and reinfection is likely to become more common [20], studying the reinfection interval is important.

To reduce infection numbers, SARS-CoV-2 vaccination in Korea began on 26 February 2021, focusing first on the elderly, immunocompromised population and healthcare workers, and then expanding to the rest of the population. Overall, 87.9% of the Korean population [21] and 87.7% of the population in Seoul have received basic vaccination (two doses of messenger ribonucleic acid (mRNA) and ChAdOx1 vaccine, one dose of Ad26.COV2.S) [22].

This study aimed to analyze the effect of SARS-CoV-2 vaccination on symptoms and severity during reinfection and identify the effects of vaccination on the reinfection interval.

## 2. Materials and Methods

### 2.1. Study Design and Cohort Construction

This was a retrospective cohort study conducted on first-infected Seoul citizens. The observation period was from 1 January 2020 to 24 February 2022. SARS-CoV-2 infection data were compiled and refined according to the Seoul Metropolitan Government’s data, and vaccine information was provided by the Korea Centers for Disease Control and Prevention (KCDC). The sampling frame was the total population aged 18 years or older residing in Seoul. The study defined pandemic periods per national genomic surveillance as follows: Pre-Delta, 1 January 2020 to 30 June 2021; Delta, 1 July to 31 December 2021; and Omicron, 1 January to 30 April 2022 [23].

### 2.2. Study Cases

Infection cases only included those confirmed positive by RT-PCR testing during the study period. Reinfection cases were defined as ≥2 positive confirmations in the same person and an interval of at least 90 days between the first infection and reinfection dates, following the National Institutes of Health and the literature [11,24,25,26]. There were some cases of third infection among the study subjects; however, only reinfection was considered in this study. The reinfection interval was defined as the interval between consecutive infections confirmed by RT-PCR testing, and not the date of symptom onset, to prevent the omission of asymptomatic cases.

All SARS-CoV-2 vaccination records are uploaded and managed in real-time through the Disease Control and Prevention Integrated Management System in South Korea. Using this system, vaccinated patients were identified as those who had completed basic vaccination [27,28] (e.g., two doses of the mRNA and ChAdOx1 vaccine, one dose for Ad26.COV2.S) against COVID-19 between 26 February 2021 and 24 February 2022. Only licensed vaccines in Korea, with proven effectiveness in preventing infection [29,30,31,32,33,34] (ChAdOx1—Oxford University/AstraZeneca vaccine, Ad26.COV2.S—Johnson & Johnson (Janssen) vaccine, BNT162b2—BioNTech (Pfizer) vaccine, and mRNA-1273—Moderna vaccine), were included. The number of people vaccinated with the mRNA type of vaccine was 957, and with the vector type of vaccine was 231, and this included vaccinations from the 1st to the 4th round (Table S1). Those who had not completed basic vaccination were considered unvaccinated (*n* = 202). The vaccine data in the KCDC were documented along with the vaccination date, vaccine type, sex, and age. The collected infection and vaccination data were integrated to confirm the precedent relationship between the two datasets (Table S2). Patients infected within 14 days after vaccination were considered unvaccinated [35,36].

### 2.3. Data Selection

This retrospective cohort study was a public health investigation and research on infectious diseases targeting Korea, legally mandatory following the Infectious Disease Prevention Act (Nos. 14,316 [37] and 30,743 [38]). The Seoul Metropolitan Government conducted COVID-19 tests free of charge for anyone who had been in contact with a COVID-19 patient or wanted to be tested for suspected COVID-19 from November 2020 to 1 October 2022 [39]. With this, the COVID-19 test rate showed an increasing trend over time, and by 24 February 2022, the number of tests exceeded 32 million (the number that most Seoul citizens had received a COVID-19 RT-PCR test) [39]. The study outcome index was reinfection, and the secondary outcome index was the reinfection interval. The endpoint for the vaccine effectiveness was defined as the occurrence of COVID-19 reinfection, and for individuals who did not experience reinfection, the endpoint was set as the end of the study period, which was 24 February 2022. Potential confounding factors included sex, age, period of variant predominance, symptoms, underlying comorbidities, vaccination, and the effect of each variable combination.

The presence or absence of symptoms and severity during infection and reinfection were investigated using the same tool [40]. This study included information on sex, age, infection date, and symptoms and severity during infection. Fifteen symptoms were recorded, and the symptoms were further analyzed in three categories: systemic, including fever (>38 °C), muscle pain, chills, headache, malaise and fatigue, allergic reaction, and dizziness; local, including cough, sputum, sore throat, rhinorrhea, gastrointestinal disorder, and chest pain; and neurological, including the loss of smell and taste. Following the KCDC guidelines [41], severity was classified by Seoul city patient management teams of doctors during occurrence into three stages: asymptomatic and mild, if the situation immediately after the first infection did not interfere with daily life and oxygen therapy was unnecessary, even if it interfered with daily life; moderate, in cases of nasal tube or mask oxygen treatment; and severe, depending on the use of non-invasive ventilation, high-flow oxygen therapy, invasive ventilation, multi-organ damage, use of extracorporeal membrane oxygenation, or continuous renal replacement therapy. The clinical symptoms and their severity were assessed following the same guidelines [41].

### 2.4. Statistical Analysis

This study used the chi-square test or Fisher’s exact test to compare differences between the vaccinated and unvaccinated groups to determine the vaccination’s effect on reducing the number of clinical symptoms at first infection and reinfection. Cochran–Mantel–Haenszel analysis was performed to confirm differences in proportions of symptoms and severity according to vaccination status. Given that severity information was unavailable in some patients, the chi-square analysis was performed by classifying these patients into two groups: asymptomatic or mild group, and moderate or severe group. To determine the effect of time of the reinfection interval by sex, age, presence of symptoms, vaccination, and underlying morbidities, Kaplan–Meier analyses were performed, and multivariate regression analysis was performed with variables showing significant results in the Kaplan–Meier analysis. Age groups were divided into 10-year intervals (18–19, 20–29, 30–39, 40–49, 50–59, 60–69, 70–79, ≤80), where the 10s group included those aged 18–19 years. All results were estimated with 95% confidence intervals (CIs), and *p* values < 0.05 indicated statistical significance. All statistical analyses were performed using the Statistical Package for the Social Sciences software (version 24.0, IBM Corp., Armonk, NY, USA).

As this is an ongoing study with analysis triggered by novel circumstances, such as the emergence of the Omicron variant and increased reinfection, the sample sizes were not calculated before its initiation. Instead, the final sample size was calculated considering the inclusion and exclusion criteria.

### 2.5. Ethical Consideration

This study used secondary data approved by the Public Institutional Review Board designated by the Ministry of Health and Welfare of the Republic of Korea (IRB Approval Number: P01-202107-21-075). Patient consent was not required in this study. The results were reported following the STROBE guidelines [42] (Table S3). All authors confirmed the accuracy and completeness of the data and the study’s originality.

## 3. Results

### 3.1. General Characteristics

Among the patients aged >17 years, 488,455 patients with SARS-CoV-2 infection con-firmed with RT-PCR testing were identified. Among these, 447,223 cases of infection were initially eligible, and 712 cases satisfying the definition of reinfection were included in the final analyses (Figure S1). Among the included patients, 43.8% were male, and the average age was 40.52 ± 16.41 years. A total of 510 individuals (71.6%) belonged to the vaccinated group, and no significant differences in gender distribution and most underlying comorbidities were observed between the vaccinated and unvaccinated groups. However, significant differences were found between the groups in terms of two underlying morbidities: hypertension and respiratory diseases. The majority (31.3%) of the patients with reinfection were in their 20s, while a minority (1.4%) were in their 80s (Table 1). The distribution of primary infection was 44.7% Pre-Delta and 55.3% Delta variants, and the distribution of reinfection was 7.3% Delta and 92.7% Omicron variants (Figure S2 and Tables S5 and S6).

This study includes patients with SARS-CoV-2 reinfection aged >17 years and who had an interval of at least 90 days between two infections between January 2020 and February 2022.

### 3.2. Descriptive Data

#### 3.2.1. Changes in Symptoms and Severity upon Reinfection According to Vaccination

In the unvaccinated group, during the reinfection compared to the first infection, the proportion of systemic symptoms decreased significantly by 23.08% (*p* < 0.001). In the vaccinated group, during the reinfection compared to the first infection, the proportion of systemic symptoms decreased significantly by 35.16%; local symptoms also significantly decreased by 11.39% during the reinfection compared to the first infection and neurological symptoms decreased by 11.03% (*p* < 0.001) (Table 2). An analysis according to vaccination status showed that the symptom reduction rate during reinfection compared to that during the first infection was significantly higher in the vaccinated than in the unvaccinated group (*p* < 0.001). The systemic symptom reduction rate, local symptom reduction rate, and neurological symptom reduction rate were significantly higher by 12.1%, 4.1%, and 5.2%, respectively (*p* < 0.05). The symptom reduction rates during reinfection for loss of smell and chills were significantly higher by 5.7% and 5.5%, respectively (*p* < 0.001) in the vaccinated group, and the symptom reduction rate for cough was significantly higher by 6.2% in the unvaccinated group (*p* = 0.022) (Table 2, Figure S3 and Table S7).

#### 3.2.2. Influencing Factors of Reinfection Interval

The average reinfection interval was 265.78 (95% CI: 253.81–277.76) days. The reinfection interval by age group increased gradually from 234.59 days for those in their 10s to 322.22 days for those in their 60s (*p* < 0.001). The reinfection interval was significantly longer in the vaccinated group than in the unvaccinated group (283.79 days vs. 220.32 days, *p* < 0.001) and was significantly shorter in the symptomatic group than in the asymptomatic group (254.29 days vs. 311.52 days, *p* < 0.001). The reinfection intervals of different sexes and underlying comorbidities were similar (Table 3). The evaluation of vaccine effectiveness by vaccine type is presented in Table S8. Nevertheless, it is essential to approach the interpretation of these results with caution due to substantial differences in vaccination timing, target populations, and the number of vaccinated individuals among various vaccines (Table S4). Multivariate regression analysis included sex, age, presence of symptoms at the first infection, and vaccination; variables with significant results in the Kaplan–Meier analysis were used. During the Pre-Delta period, the reinfection interval increased significantly by 0.838 days for every 10-year increase in age and was significantly higher by 30.964 days in the vaccinated group than in the unvaccinated group (all *p* < 0.05). However, during the Delta period, reinfection intervals did not change significantly, with or without vaccination (Table 4).

## 4. Discussion

This paired comparison study was conducted to confirm the effect of vaccination on the symptoms, severity, and reinfection interval of SARS-CoV-2 in the same person. In this study, patients who received vaccination showed a higher proportion of asymptomatic cases and milder symptoms during reinfection compared to those who were unvaccinated. Additionally, the reinfection interval was observed to be prolonged.

The reduction rate of symptoms upon reinfection compared to the first infection was significantly higher by 0.9% in the vaccinated group, consistent with previous findings that vaccination is significantly associated with symptomatic SARS-CoV-2 infection reduction [43]. Further, the reduction in systemic, local, and neurological symptoms was significantly higher in the vaccinated group than in the unvaccinated group. In the vaccinated group, the reduction rates of fever, myalgia, chills, headache, malaise and fatigue, and loss of smell and taste at reinfection were significantly higher than those in the unvaccinated group. The difference in proportions of moderate or severe cases according to vaccination status was insignificant.

However, to the best of our knowledge, previous studies have not compared the symptoms and severity between the first and second infections in the same patients in community-based cohort, and changes in detailed clinical symptoms of reinfection according to vaccination status have also not been studied to date. Nevertheless, the results of this study, conducted on the same patients, support the previous studies indicating that vaccination reduces the severity of reinfection [44,45]. However, a cohort study conducted in Denmark revealed that two vaccine doses offered merely a restricted and brief shield against SARS-CoV-2 infection with Omicron. Consequently, further ongoing research is warranted [46].

Vaccination status, presence of symptoms during the first infection, date of the first infection, and age difference significantly affected the reinfection interval. The reinfection interval gradually increased with age until the 60s. It was 63.47 days longer for the vaccinated group than that for the unvaccinated group, and 57.23 days longer for the asymptomatic group than for the symptomatic group. In multivariate regression analysis, during the Pre-Delta period, the reinfection interval increased significantly by 0.838 days for each 10-year increase in age and was significantly longer by 30.964 days for the vaccinated group. During the Delta period, the reinfection interval decreased significantly by 0.223 days for each 10-year increase in age. Vaccination during the Pre-Delta period significantly affected the reinfection interval, but vaccination during the Delta period did not. The reason for the impact of the reinfection interval during the pre-delta stage is believed to be twofold. Firstly, the SARS-CoV-2 infectivity became stronger during the Delta stage [47,48,49], and secondly, the vaccine effectiveness was reduced in preventing SARS-CoV-2 infections during the Delta stage [50,51]. We were able to identify almost all of the secondary infections, regardless of the vaccination status, as we observed all subjects under the same conditions. Therefore, even if there were more cases of infections, there might have been differences in the effectiveness of the vaccine during the Pre-Delta and Delta stages. Furthermore, the effectiveness of the vaccine has continuously varied according to viral mutations [51,52]. In addition, this is identical to previous studies that showed that regardless of whether infection occurred before or after vaccination, the gradual decline in antibody titers according to hybrid immunity resulted in a decline in immunity, eventually losing protection against SARS-CoV-2 variant strains [4,11,14,53]. While many studies, including this research, support the effectiveness of the vaccine [1,2,3,4,10,11], there are also studies that suggest the vaccine afforded less protection, and effectiveness was not demonstrated [54].

To the best of our knowledge, no previous study has analyzed the influence of various factors on the reinfection interval through multivariate regression analysis. Also, in a previous study on a single variable, no relationship between age at first infection and reinfection was found [55]. By adding various variables to the results of previous studies, the current study confirmed that age can affect the reinfection interval. A previous study found that vaccination increased neutralizing antibodies that suppressed infection and increased immunity [10], supporting the hypothesis that vaccinations led to longer intervals for reinfection in the Pre-Delta period. Nevertheless, the efficacy of these vaccines has not been firmly established through long-term studies, and it cannot fully account for the impact of unmeasured factors. Consequently, akin to the situation observed with the previous BCG vaccine [56], the vaccine’s effectiveness is beneficial rather than fully sufficient. However, there are few studies on how sex, symptoms at the first infection, underlying comorbidity, and vaccination affect the reinfection interval, and further research is needed.

This study had some limitations. First, as a study targeting Seoul citizens, it did not represent all reinfection cases. Nevertheless, a cohort was established for all patients infected with SARS-CoV-2, and reinfection cases were observed. Furthermore, since the same person was examined for symptoms and severity was measured with the same tool, as many confounding factors as possible were eliminated. Therefore, reinfected individuals can be considered representative by establishing an all-inclusive cohort within the community. Second, when analyzing the reinfection interval, Kaplan–Meier and Cox proportional hazards models were considered, but the vaccination effect decreased over time and did not satisfy the assumption of Cox proportional hazards analysis. Therefore, we used multivariate regression analysis, divided by two peaks according to the duration of the outbreak of the variant virus during the first infection, instead of Cox proportional hazards analysis. Third, since the SARS-CoV-2 infection date was based on the date of confirmation in the RT-PCR test, it may be unclear when exactly the infection occurred. However, since the SARS-CoV-2 infection date was determined by applying the same criteria to all infected patients, there would be no difference between vaccinated and unvaccinated patients. Additionally, this study is an observational epidemiological study without the use of placebo vaccines, which limited our ability to exclude the influence of nonidentical infection pressure. However, the factors influencing the vaccine’s efficacy were symmetrically distributed between the vaccine and non-vaccine groups, and there was no evidence of reverse surveillance bias in disease monitoring. Lastly, although the non-specific effects of COVID-19 vaccination have been discussed and acknowledged [57,58], the assessment of the vaccine’s non-specific effects was not within the primary scope of this study, as our primary objective was not to confirm their impact. Moreover, the potential for false positives and the challenge of differentiating between various respiratory infections also could potentially affect the interpretation of the results.

## 5. Conclusions

As COVID-19 spreads, it is important to understand the patterns and characteristics of reinfection cases, confirm vaccine protection, and encourage unvaccinated infected persons to become vaccinated. It was confirmed that vaccination relieved the clinical symptoms during reinfection. In particular, systemic and neurological symptoms were significantly reduced during reinfection in the vaccinated group. The factors influencing the reinfection interval were age, the presence of symptoms during the first infection, and vaccination. In the Pre-Delta period, age and vaccination had a significant effect on the reinfection interval, and in the Delta period, age had a significant effect.

## Figures and Tables

**Table 1:** General characteristics of the study population based on vaccination status.

Characteristics	Total		Unvaccinated		Vaccinated		*p*-Value ^a^
	No.	%	No.	%	No.	%	
Total	712	100.0	202	28.4	510	71.6	
Age							
Age (years), mean (SD)	40.52	16.4	36.91	14.84	41.95	16.80	<0.001
18–19	22	3.1	13	6.4	9	1.8	0.002
20–29	223	31.3	67	33.2	156	30.6	
30–39	135	19.0	48	23.8	87	17.1	
40–49	131	18.4	30	14.9	101	19.8	
50–59	80	11.2	22	10.9	58	11.4	
60–69	82	11.5	16	7.9	66	12.9	
70–79	29	4.1	4	2.0	25	4.9	
≥80	10	1.4	2	1.0	8	1.6	
Sex							
Female	400	56.2	110	54.5	290	56.9	0.31
Male	312	43.8	92	45.5	220	43.1	
Underlying morbidity							
Total	162	22.8	43	21.3	119	23.3	
Having an underlying morbidity, mean (SD)	1.46	0.79	1.26	0.64	1.53	0.83	0.10
Allergic disease	2	1.2	0	0.0	2	1.7	0.51
Cancer	6	3.7	1	2.3	5	4.2	0.46
Cardiovascular system disease	11	6.8	1	2.3	10	8.4	0.13
Cerebrovascular disease	6	3.7	2	4.7	4	3.4	0.55
Diabetes mellitus	39	24.1	8	18.6	31	26.1	0.18
Endocrine disease	7	4.3	3	7.0	4	3.4	0.32
Hypertension	62	38.3	11	25.6	51	42.9	0.03
Immunocompromised disease	3	1.9	2	4.7	1	0.8	0.16
Kidney disease	6	3.7	3	7.0	3	2.5	0.23
Liver disease	2	1.2	0	0.0	2	1.7	0.51
Mental disorder	14	8.6	2	4.7	12	10.1	0.19
Respiratory system disease	28	17.3	13	30.2	15	12.6	0.03

^a^ *p*-value from chi-square test or Fisher’s exact test.

**Table 2:** Changes in apparent symptom frequencies between the first infection and reinfection of COVID-19 according to vaccination status.

	Unvaccinated (*n* = 202)				*p* Value	Vaccinated (*n* = 510)				*p* Value	Difference in Proportions of Symptoms According to Vaccination Status ^a^		
	1st Infection		2nd Infection			1st Infection		2nd Infection			Diff %	Diff *p* Value	OR (95% CI ^b^)
	No.	%	No.	%		No.	%	No.	%				
Symptom													
No	36	17.82	79	39.11	<0.001	107	20.98	220	43.14	<0.001	−0.87	<0.001	0.35 (0.27–0.44)
Yes	166	82.18	123	60.89		403	79.02	290	56.86				
Symptom type													
Systemic symptom	95	57.23	42	34.15	<0.001	232	57.57	65	22.41	<0.001	−12.08	<0.001	0.26 (0.20–0.34)
Fever	66	39.76	24	19.51	<0.001	139	34.49	32	11.03	0.001	−3.21	<0.001	0.28 (0.20–0.38)
Myalgia	51	30.72	20	16.26	0.003	110	27.30	34	11.72	<0.001	−1.12	<0.001	0.38 (0.27–0.53)
Chills	38	22.89	17	13.82	0.04	92	22.83	24	8.28	<0.001	−5.48	<0.001	0.37 (0.25–0.54)
Headache	46	27.71	24	19.51	0.07	95	23.57	38	13.10	<0.001	−2.27	<0.001	0.53 (0.38–0.74)
Malaise and fatigue	5	3.01	4	3.25	0.58	28	6.95	6	2.07	0.002	−5.12	0.02	0.40 (0.20–0.83)
Allergic	0	0.00	2	1.63	0.18	1	0.25	2	0.69	0.38	−1.19	0.21	5.56 (0.62–50.0)
Dizziness	1	0.60	1	0.81	0.67	5	1.24	1	0.34	0.21	−1.11	0.54	0.46 (0.09–2.28)
Local symptom	112	67.47	74	60.16	0.12	278	68.98	167	57.59	<0.001	−4.08	0.001	0.64 (0.49–0.84)
Cough	83	50.00	47	38.21	0.03	163	40.45	101	34.83	0.08	6.17	0.02	0.73 (0.56–0.95)
Sputum	41	24.70	27	21.95	0.34	86	21.34	69	23.79	0.25	5.20	0.79	1.05 (0.78–1.42)
Sore throat	68	40.96	53	43.09	0.40	150	37.22	117	40.34	0.23	0.99	0.41	1.13 (0.87–1.46)
Rhinorrhea	11	6.63	16	13.01	0.05	32	7.94	20	6.90	0.36	−7.42	0.59	1.17 (0.73–1.85)
Gastro symptom	2	1.20	1	0.81	0.61	6	1.49	4	1.38	0.59	0.28	0.98	0.86 (0.28–2.65)
Chest pain	1	0.60	1	0.81	0.67	4	0.99	3	1.03	0.62	−0.17	0.85	1.11 (0.30–4.14)
Neurological symptom	15	9.04	4	3.25	0.08	50	12.41	4	1.38	<0.001	−5.24	<0.001	0.15 (0.07–0.32)
Loss of smell	6	3.61	3	2.44	0.42	32	7.94	3	1.03	<0.001	−5.74	<0.001	0.21 (0.09–0.49)
Loss of taste	11	6.63	2	1.63	0.04	29	7.20	2	0.69	<0.001	−1.51	<0.001	0.13 (0.05–0.36)
Severity ^c^	(*n* = 193)		(*n* = 78)			(*n* = 495)		(*n* = 153)					
Mild or Lower	188	97.41	74	94.87	0.24	483	97.58	150	98.04	0.51	−3.00	0.86	1.21 (0.49–2.98)
Moderate or Severe	5	2.59	4	5.13		12	2.42	3	1.96				

Data are presented as *n* or %. ^a^ “Difference in proportions of symptoms according to vaccination status” means differences in proportions of a decrease in symptoms when confirming the comparison of first and second infection symptoms and the type of symptoms in the same person in comparison with the vaccinated group and the unvaccinated group, respectively. Cochran–Mantel–Haenszel analysis was performed to confirm the difference in proportions of symptoms and severity according to vaccination status. ^b^ CI, confidence interval. ^c^ Severity classified into two groups: asymptomatic or mild, and moderate or severe. Patients had been residents of Seoul since 1 January 2020. All patients were reinfected with SARS-CoV-2.

**Table 3:** Distribution of the intervals for reinfection by age, underlying comorbidities, symptoms at the first infection, period of variant predominance, and vaccination status.

Reinfection Interval	No.	Median	IQR ^a^	Mean (95% CI ^b^)	*p*-Value ^c^
Total	712	164.50	129.00–419.00	265.78 (253.81–277.76)	
Age group, years					
18–19	22	147.00	134.00–358.25	234.59 (176.54–292.65)	0.003
20–29	223	157.00	129.00–387.00	237.83 (218.03–257.63)	
30–39	135	154.00	121.00–397.00	242.21 (217.21–267.22)	
40–49	131	170.00	128.00–425.00	279.54 (250.60–308.49)	
50–59	80	308.00	132.50–430.00	301.14 (262.24–340.04)	
60–69	82	361.00	148.00–438.50	322.22 (285.46–358.97)	
70–79	29	152.00	120.50–452.00	284.48 (219.27–349.69)	
≥80	10	186.00	110.25–466.75	295.90 (179.57–412.23)	
Vaccination					
Unvaccinated	202	141.00	119.00–323.25	220.32 (199.25–241.38)	<0.001
Vaccinated	510	218.00	133.75–424.00	283.79 (269.63–297.96)	
Presence of symptoms (1st infection)					
No	143	356.00	144.00–432.00	311.52 (284.57–338.46)	0.005
Yes	569	158.00	127.60–413.00	254.29 (241.11–267.47)	
Period of variant predominance					
Pre-Delta period	319	425.00	376.00–463.00	428.44 (417.34–439.53)	<0.001
Delta period	393	133.00	113.00–151.00	133.76 (131.05–136.47)	
Sex					
Female	400	165.00	129.50–421.75	268.48 (252.18–284.78)	0.22
Male	312	161.00	128.00–416.00	262.33 (244.77–279.89)	
Presence of underlying morbidity					
No	550	161.00	131.00–414.00	260.31 (246.98–273.63)	0.16
Yes	162	221.00	122.75–431.00	284.38 (257.78–310.99)	

Data are presented as *n* or %. ^a^ IQR, interquartile range. ^b^ CI, confidence interval. ^c^ Kaplan–Meier analyses were performed to determine the effect of time of the reinfection interval by sex, age, presence of symptoms, vaccination, and underlying morbidities. Patients had been residents of Seoul since 1 January 2020. All patients were reinfected with SARS-CoV-2.

**Table 4:** Multivariate regression analysis of the reinfection interval using sex, age, presence of symptoms at the first infection, and vaccination as variables.

	Coefficient	95% CI ^a^		Standard Error	*p*-Value ^b^
Pre-Delta period					
Sex	−12.446	−34.685	9.794	11.303	0.23
Age	0.838	0.172	1.504	0.339	0.01
Presence of symptoms at the first infection	7.274	−17.792	32.34	12.739	0.57
Vaccination	30.964	3.594	58.335	13.911	0.03
Delta period					
Sex	−0.269	−5.742	5.205	2.783	0.92
Age	−0.223	−0.397	−0.051	0.088	0.01
Presence of symptoms at the first infection	−3.839	−11.698	4.019	3.997	0.34
Vaccination	5.141	−0.67	10.953	2.956	0.08

^a^ CI, confidence interval. ^b^ Multivariate regression analysis was performed with significant variables in the Kaplan–Meier analysis. Patients had been residents of Seoul since 1 January 2020. All patients were reinfected with SARS-CoV-2.

## Data Availability

The data used in this study are unavailable due to privacy restrictions.

## Supplementary Materials

The following supporting information can be downloaded at: https://www.mdpi.com/article/10.3390/vaccines11091408/s1, Figure S1: Flow chart for the identification of COVID-19 reinfection; Figure S2: Distribution of the date of first infection and reinfection including vaccination information by the variant virus epidemic period; Figure S3: Reduced proportion of apparent symptoms between the first infection and reinfection of SARS-CoV-2 according to vaccination status; Table S1: Number of persons vaccinated with a particular type of vaccine; Table S2: Classification of vaccinated and non-vaccinated groups according to the order of COVID-19 vaccination and infection; Table S3: STROBE Statement—Checklist of items that should be included in reports of cohort studies; Table S4: General characteristics of the vaccinated population based on the vaccination type; Table S5: Comparison of characteristics of primary and secondary vaccination by the variant virus epidemic period on a weekly basis; Table S6: Comparison of characteristics of primary and secondary vaccination during the variant virus epidemic period on a monthly basis; Table S7: Comparison of differences in symptoms after first confirmation and reconfirmation according to vaccination status (present/absent); Table S8: Distribution of the intervals for reinfection by type of vaccine.
