# Supplementary material for: Effects of Severe Acute Respiratory Syndrome Coronavirus Vaccination on Reinfection: A Community-Based Retrospective Cohort Study
Source: Vaccines (Basel). 2023 Aug 23;11(9):1408. doi: 10.3390/vaccines11091408 (PMC10535171; doi:10.3390/vaccines11091408)
Supplement: Supplementary file 1 [file vaccines-11-01408-s001.zip › vaccines-2520044-supplementary.pdf]

Supplementary Materials

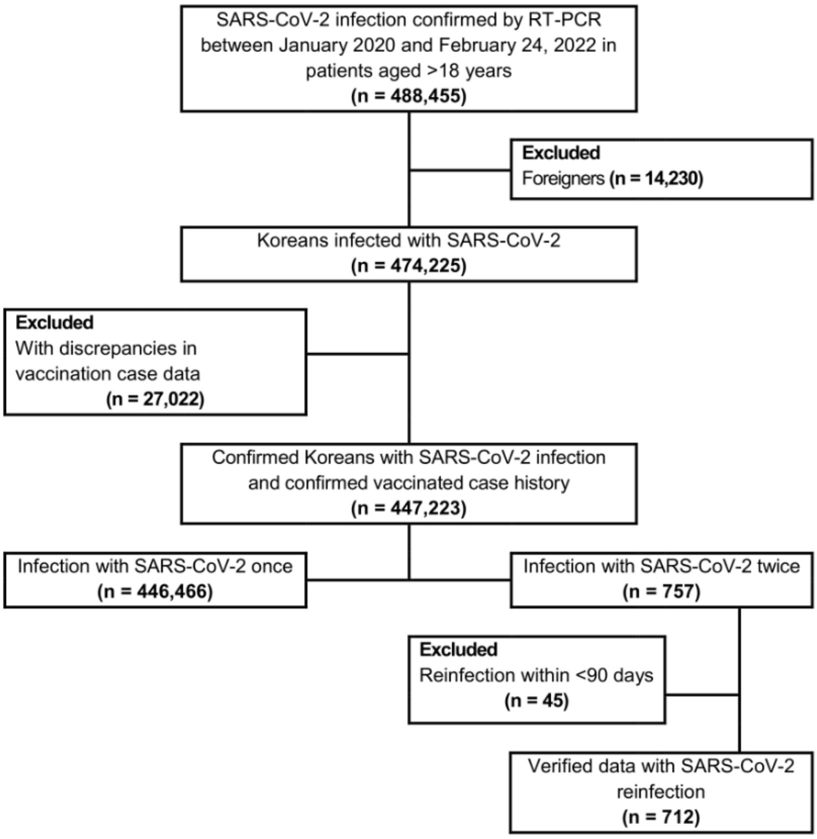

Figure S1. Flow chart for the identification of COVID-19 reinfection.

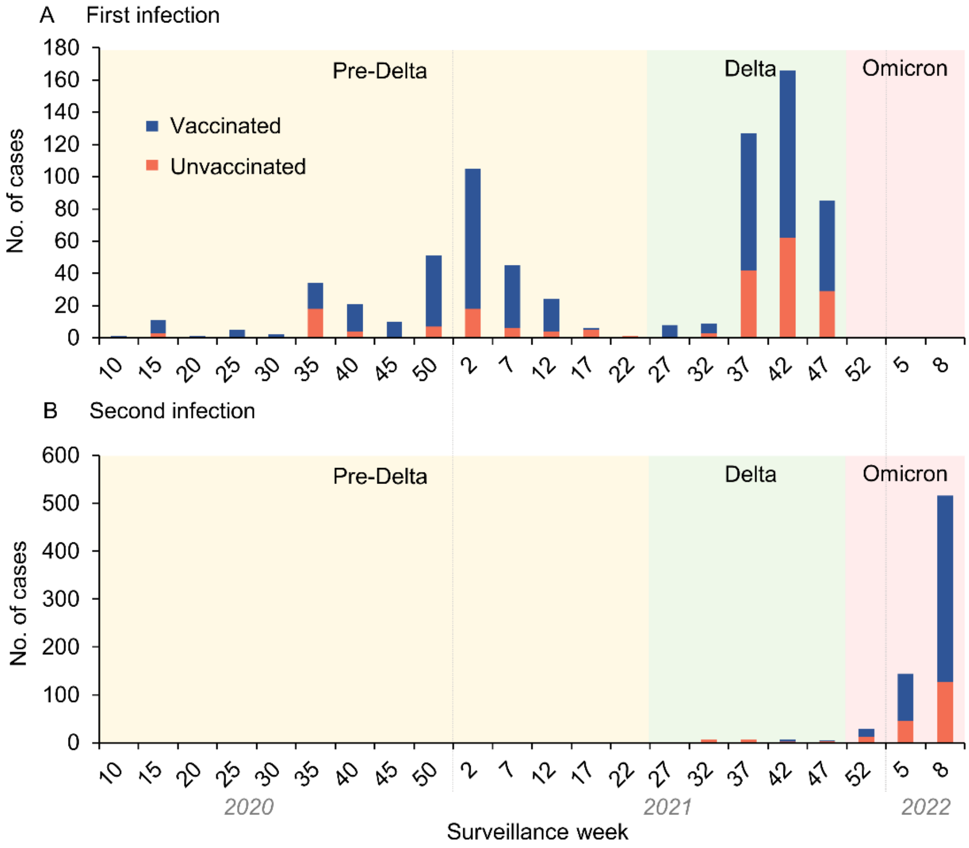

Figure S2. Distribution of the date of first infection and reinfection including vaccination information by the variant virus epidemic period

A. Distribution of the date of the first infection including vaccination information by the Pre-Delta and Delta periods
B. Distribution of the date of reinfection including vaccination information by the Delta and Omicron periods

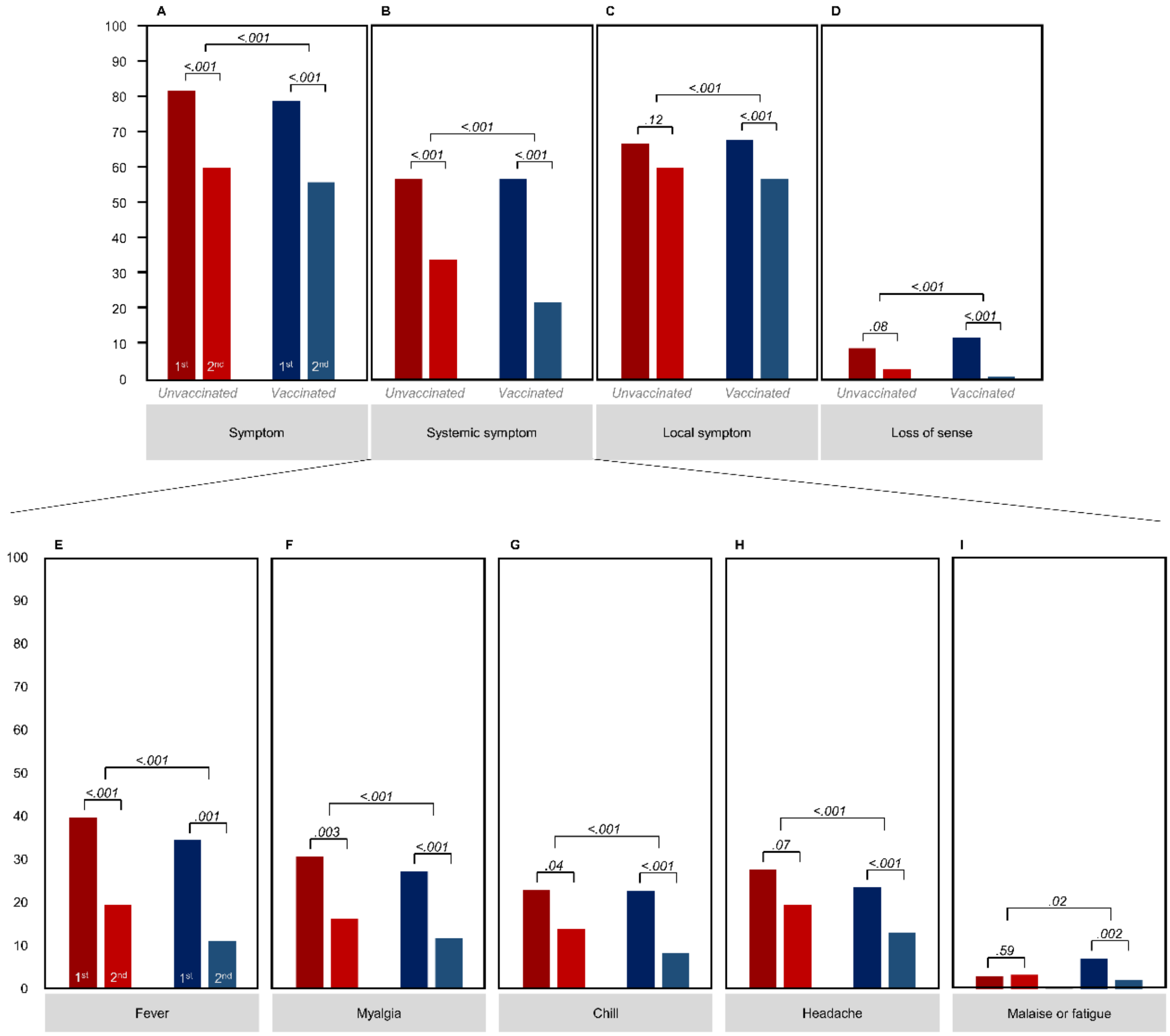

Figure S3. Reduced proportion of apparent symptoms between the first infection and reinfection of SARS-CoV-2 according to vaccination status.

Table S1. Number of persons vaccinated with a particular type of vaccine

	First vaccination No.	Second vaccination No.	Third vaccination No.
ChAdOx1	112	94	
Ad26.COV2.S	25		
BNT162b2	325	345	146
mRNA-1273	48	46	47

Table S2. Classification of vaccinated and non-vaccinated groups according to the order of COVID-19 vaccination and infection

	**Groups determined by the order of COVID-19 vaccination and infected COVID-19**					**No.**	**Category**
1	1st Infection	Reinfection				202	Unvaccinated
2	1st Infection	Vaccination 1	Vaccination 2	Reinfection		222	Vaccinated
3	1st Infection	Vaccination 1	Vaccination 2	Vaccination 3	Reinfection	117	Vaccinated
4	Vaccination 1	1st Infection	Vaccination 2	Reinfection		56	Vaccinated
5	Vaccination 1	1st Infection	Vaccination 2	Vaccination 3	Reinfection	12	Vaccinated
6	Vaccination 1	Vaccination 2	1st infection	Reinfection		39	Vaccinated
7	Vaccination 1	Vaccination 2	1st Infection	Vaccination 3	Reinfection	64	Vaccinated

Table S3. STROBE Statement—Checklist of items that should be included in reports of cohort studies–

	Item No	Recommendation	Page No.
**Title and abstract**	1	(*a*) Indicate the study's design with a commonly used term in the title or the abstract	1
		(*b*) Provide in the abstract an informative and balanced summary of what was done and what was found	1
**Introduction**			
Background/rationale	2	Explain the scientific background and rationale for the investigation being reported	1–2
Objectives	3	State specific objectives, including any prespecified hypotheses	2
**Methods**			
Study design	4	Present key elements of study design early in the paper	2
Setting	5	Describe the setting, locations, and relevant dates, including periods of recruitment, exposure, follow-up, and data collection	2–3
Participants	6	(*a*) *Cohort study* - Give the eligibility criteria, and the sources and methods of selection of participants. Describe methods of follow-up	2–3
		(*b*) *Cohort study* - For matched studies, give matching criteria and number of exposed and unexposed	4
Variables	7	Clearly define all outcomes, exposures, predictors, potential confounders, and effect modifiers. Give diagnostic criteria, if applicable	2–3
Data sources/ measurement	8*	For each variable of interest, give sources of data and details of methods of assessment (measurement). Describe comparability of assessment methods if there is more than one group	2–3, Table A1
Bias	9	Describe any efforts to address potential sources of bias	2–3
Study size	10	Explain how the study size was arrived at	4
Quantitative variables	11	Explain how quantitative variables were handled in the analyses. If applicable, describe which groupings were chosen and why	2–3, Table A2
Statistical methods	12	(*a*) Describe all statistical methods, including those used to control for confounding	3
		(*b*) Describe any methods used to examine subgroups and interactions	3
		(*c*) Explain how missing data were addressed	-
		(*d*) *Cohort study* - If applicable, explain how loss to follow-up was addressed	-

		(*e*) Describe any sensitivity analyses	-
**Results**			
Participants	13*	(a) Report numbers of individuals at each stage of the study—eg numbers potentially eligible, examined for eligibility, confirmed eligible, included in the study, completing follow-up, and analysed	4
		(b) Give reasons for non-participation at each stage	4
		(c) Consider use of a flow diagram	-
Descriptive data	14*	(a) Give characteristics of study participants (eg demographic, clinical, social) and information on exposures and potential confounders	4, Table A4
		(b) Indicate number of participants with missing data for each variable of interest	4
		(c) Summarise follow-up time (eg, average and total amount)	4, Table A5 and A6, Figure A1
Outcome data	15*	*Cohort study* - Report numbers of outcome events or summary measures over time	4, Table 1, Figure A1
Main results	16	(*a*) Give unadjusted estimates and, if applicable, confounder-adjusted estimates and their precision (eg, 95% confidence interval). Make clear which confounders were adjusted for and why they were included	4–9, Tables 1–4, Tables A7
		(*b*) Report category boundaries when continuous variables were categorized	3
		(*c*) If relevant, consider translating estimates of relative risk into absolute risk for a meaningful time period	4–9
Other analyses	17	Report other analyses done—eg analyses of subgroups and interactions, and sensitivity analyses	4–9, Figure A2, Table A8
**Discussion**			
Key results	18	Summarise key results with reference to study objectives	9-10
Limitations	19	Discuss limitations of the study, taking into account sources of potential bias or imprecision. Discuss both direction and magnitude of any potential bias	9
Interpretation	20	Give a cautious overall interpretation of results considering objectives, limitations, multiplicity of analyses, results from similar studies, and other relevant evidence	8–9
Generalisability	21	Discuss the generalisability (external validity) of the study results	8–10
**Other information**			
Funding	22	Give the source of funding and the role of the funders for the present study and, if applicable, for the original study on which the present article is based	10

*Give information separately for exposed and unexposed groups.

Note: An Explanation and Elaboration article discusses each checklist item and gives methodological background and published examples of transparent reporting. The STROBE checklist is best used in conjunction with this article (freely available on the Web sites of PLoS Medicine at http://www.plosmedicine.org/, Annals of Internal Medicine at http://www.annals.org/, and Epidemiology at http://www.epidem.com/). Information on the STROBE Initiative is available at http://www.strobe-statement.org.

Table S4. General characteristics of the vaccinated population based on the vaccination type.

Characteristics	Total		Mix†		mRNA‡		Vector§		P value[a]
	No.	%	No.	%	No.	%	No.	%	
Total	510	100.0	107	20.98	373	73.14	30	5.88	
Age									
Age (years), mean (SD)	41.95	16.80	52.32	15.20	37.93	15.55	54.93	14.68	<.001
18–19	9	1.8	0	0.00	9	2.41	0	0.00	<.001
20–29	156	30.6	6	5.61	148	39.68	2	6.67	
30–39	87	17.1	24	22.43	57	15.28	6	20.00	
40–49	101	19.8	17	15.89	83	22.25	1	3.33	
50–59	58	11.4	7	6.54	48	12.87	3	10.00	
60–69	66	12.9	42	39.25	8	2.14	16	53.33	
70–79	25	4.9	11	10.28	13	3.49	1	3.33	
≥80	8	1.6	0	0.00	7	1.88	1	3.33	
Sex									
Female	290	56.9	53	49.53	227	60.86	10	33.33	0.003
Male	220	43.1	54	50.47	146	39.14	20	66.67	
Underlying morbidity									
Total	119	23.3	31	28.97	60	16.09	11	36.67	
Having an underlying morbidity, mean (SD)	1.53	0.83	1.29	0.46	1.4	0.79	1.45	0.82	0.001
Allergic disease	2	1.7	1	3.23	1	0.27	0	0.00	0.585
Cancer	5	4.2	0	0.00	3	0.80	2	6.67	0.004
Cardiovascular system disease	10	8.4	1	3.23	7	1.88	2	6.67	0.132
Cerebrovascular disease	4	3.4	2	6.45	2	0.54	0	0.00	0.341
Diabetes mellitus	31	26.1	9	29.03	19	5.09	3	10.00	0.292

Endocrine disease	4	3.4	2	6.45	2	0.54	0	0.00	0.341
Hypertension	51	42.9	19	61.29	26	6.97	6	20.00	0.001
Immunocompromised disease	1	0.8	0	0.00	1	0.27	0	0.00	0.832
Kidney disease	3	2.5	1	3.23	2	0.54	0	0.00	0.813
Liver disease	2	1.7	0	0.00	1	0.27	1	3.33	0.027
Mental disorder	12	10.1	2	6.45	10	2.68	0	0.00	0.605
Respiratory system disease	15	12.6	3	9.68	10	2.68	2	6.67	0.460

[a] P value from chi-square test or Fisher's exact test.
†Received vaccine only of mRNA (BNT162b2 (tozinameran, Pfizer-BioNtech) or mRNA-1273 (lasomeran, Moderna)) or Vector (Jcovden (Ad26.COV2.S, previously COVID-19 Vaccine Janssen) or ChAdOx1-S [recombinant] COVID-19 vaccine (AstraZeneca, ChAdOx1-S)) vaccines before February 24, 2022.
‡Received vaccine only of mRNA (BNT162b2 (tozinameran, Pfizer-BioNtech) or mRNA-1273 (lasomeran, Moderna)) vaccines before February 24, 2022.
§Received vaccine only of Vector (Jcovden (Ad26.COV2.S, previously COVID-19 Vaccine Janssen) or ChAdOx1-S [recombinant] COVID-19 vaccine (AstraZeneca, ChAdOx1-S)) vaccines before February 24, 2022.

Table S5. Comparison of characteristics of primary and secondary vaccination during the variant virus epidemic period on a weekly basis

**Characteristics (n = 712)**	**1st infection day**						**2nd infection day**					
	**Unvaccinated (*n* = 202)**		**Vaccinated (*n* = 510)**		**Total**		**Unvaccinated (*n* = 202)**		**Vaccinated (*n* = 510)**		**Total**	
	**No.**	**%**	**No.**	**%**	**No.**	**%**	**No.**	**%**	**No.**	**%**	**No.**	**%**
Surveillance week												
Pre Delta period												
Week 10 of 2020	0	0	1	0.2	1	0.14	0	0	0	0	0	0.00
Week 11–15 of 2020	3	1.49	8	1.57	11	1.54	0	0	0	0	0	0.00
Week 16—20 of 2020	0	0	1	0.2	1	0.14	0	0	0	0	0	0.00
Week 21—25 of 2020	0	0	5	0.98	5	0.70	0	0	0	0	0	0.00
Week 26–30 of 2020	0	0	2	0.39	2	0.28	0	0	0	0	0	0.00
Week 31–35 of 2020	18	8.91	16	3.14	34	4.78	0	0	0	0	0	0.00
Week 36–40 of 2020	4	1.98	17	3.33	21	2.95	0	0	0	0	0	0.00

Week 41–45 of 2020	0	0	10	1.96	10	1.40	0	0	0	0	0	0.00
Week 46–50 of 2020	7	3.47	44	8.63	51	7.16	0	0	0	0	0	0.00
Week 51 of 2020—week 2 of 2021	18	8.91	87	17.06	105	14.75	0	0	0	0	0	0.00
Week 3–7 of 2021	6	2.97	39	7.65	45	6.32	0	0	0	0	0	0.00
Week 8–12 of 2021	4	1.98	20	3.92	24	3.37	0	0	0	0	0	0.00
Week 13–17 of 2021	5	2.48	1	0.2	6	0.84	0	0	0	0	0	0.00
Week 18–22 of 2021	1	0.5	0	0	1	0.14	0	0	0	0	0	0.00
Week 10 of 2020	0	0	1	0.2	1	0.14	0	0	0	0	0	0.00
Week 11–15 of 2020	3	1.49	8	1.57	11	1.54	0	0	0	0	0	0.00
Week 16—20 of 2020	0	0	1	0.2	1	0.14	0	0	0	0	0	0.00
Week 21—25 of 2020	0	0	5	0.98	5	0.70	0	0	0	0	0	0.00
Week 26–30 of 2020	0	0	2	0.39	2	0.28	0	0	0	0	0	0.00
Pre Delta and Delta period												
Week 23–27 of 2021[a]	0	0	8	1.57	8	1.12	0	0	0	0	0	0.00
Delta period												
Week 28–32 of 2021	3	1.49	6	1.18	9	1.26	6	2.97	0	0	6	0.84
Week 33–37 of 2021	42	20.79	85	16.67	127	17.84	6	2.97	1	0.2	7	0.98
Week 38–42 of 2021	62	30.69	104	20.39	166	23.31	2	0.99	4	0.78	6	0.84
Week 43–47 of 2021	29	14.36	56	10.98	85	11.94	3	1.49	2	0.39	5	0.70
Delta and Omicron period												
Week 48–52 of 2021[b]	0	0	0	0	0	0.00	12	5.94	16	3.14	28	3.93
Omicron period												
Week 1–5 of 2022	0	0	0	0	0	0.00	46	22.77	98	19.22	144	20.22
Week 6–8 of 2022	0	0	0	0	0	0.00	127	62.87	389	76.27	516	72.47

[a] The 26th week of 2021 covered June 27 to 30 (Pre-delta period) and July 1 to 3 (Delta period). And the 27th week of 2021 covered July 4 to 10(Delta period).

[b] The 52nd week of 2021 covered December 26 to 31 (Delta period) and January 1 (Omicron period).

Table S6. Comparison of characteristics of primary and secondary vaccination during the variant virus epidemic period on a monthly basis

Characteristics (n = 712)	1st infection day						2nd infection day					
	Unvaccinated (*n* = 202)		Vaccinated (*n* = 510)		Total		Unvaccinated (*n* = 202)		Vaccinated (*n* = 510)		Total	
	No.	%	No.	%	No.	%	No.	%	No.	%	No.	%
Surveillance month												
Pre Delta period												
202003	2	0.99	7	1.37	9	1.26	0	0.00	0	0.00	0	0.00
202004	1	0.50	2	0.39	3	0.42	0	0.00	0	0.00	0	0.00
202005	0	0.00	1	0.20	1	0.14	0	0.00	0	0.00	0	0.00
202006	0	0.00	6	1.18	6	0.84	0	0.00	0	0.00	0	0.00
202007	0	0.00	1	0.20	1	0.14	0	0.00	0	0.00	0	0.00
202008	21	10.40	17	3.33	38	5.34	0	0.00	0	0.00	0	0.00
202009	1	0.50	16	3.14	17	2.39	0	0.00	0	0.00	0	0.00
202010	0	0.00	6	1.18	6	0.84	0	0.00	0	0.00	0	0.00
202011	2	0.99	26	5.10	28	3.93	0	0.00	0	0.00	0	0.00
202012	20	9.90	82	16.08	102	14.33	0	0.00	0	0.00	0	0.00
202101	4	1.98	39	7.65	43	6.04	0	0.00	0	0.00	0	0.00
202102	5	2.48	33	6.47	38	5.34	0	0.00	0	0.00	0	0.00
202103	4	1.98	14	2.75	18	2.53	0	0.00	0	0.00	0	0.00
202104	5	2.48	1	0.20	6	0.84	0	0.00	0	0.00	0	0.00
202105	1	0.50	0	0.00	1	0.14	0	0.00	0	0.00	0	0.00
202106	0	0.00	1	0.20	1	0.14	0	0.00	0	0.00	0	0.00
Delta period												
202107	3	1.49	13	2.55	16	2.25	3	1.49	0	0.00	3	0.42
202108	1	0.50	3	0.59	4	0.56	4	1.98	0	0.00	4	0.56

202109	71	35.15	138	27.06	209	29.35	6	2.97	4	0.78	10	1.40
202110	39	19.31	63	12.35	102	14.33	2	0.99	2	0.39	4	0.56
202111	22	10.89	41	8.04	63	8.85	2	0.99	2	0.39	4	0.56
202112	0	0.00	0	0.00	0	0.00	12	5.94	15	2.94	27	3.79
Omicron period												
202201	0	0.00	0	0.00	0	0.00	30	14.85	67	13.14	97	13.62
202202	0	0.00	0	0.00	0	0.00	143	70.79	420	82.35	563	79.07

Table S7. Comparison of differences in symptoms after first confirmation and reconfirmation according to vaccination status (present/absent)

**Characteristics (n=712)**	**No. (%)**		***P value*[a]**	**No. (%)**		***P value*[a]**
	**1st infection symptom**			**2nd infection symptom**		
	**Unvaccinated (*n* = 202)**	**Vaccinated (*n* = 510)**		**Unvaccinated (*n* = 202)**	**Vaccinated (*n* = 510)**	
Symptom						
No	36 (17.8)	107 (21)	0.20	79 (39.1)	220 (43.1)	0.19
Yes	166 (82.2)	403 (79)		123 (60.9)	290 (56.9)	
Symptom type						
Fever	66 (39.8)	139 (34.5)	0.14	24 (19.5)	32 (11)	0.02
Cough	83 (50)	163 (40.4)	0.02	47 (38.2)	101 (34.8)	0.29
Sputum	41 (24.7)	86 (21.3)	0.22	27 (22)	69 (23.8)	0.39
Sore throat	68 (41)	150 (37.2)	0.23	53 (43.1)	117 (40.3)	0.34
Myalgia	51 (30.7)	110 (27.3)	0.23	20 (16.3)	34 (11.7)	0.14
Chill	38 (22.9)	92 (22.8)	0.53	17 (13.8)	24 (8.3)	0.06
Headache	46 (27.7)	95 (23.6)	0.18	24 (19.5)	38 (13.1)	0.07
Pneumonia	4 (2.4)	15 (3.7)	0.31	4 (3.3)	5 (1.7)	0.26
Malaise and fatigue	5 (3)	28 (6.9)	0.05	4 (3.3)	6 (2.1)	0.34
Rhinorrhea	11 (6.6)	32 (7.9)	0.37	16 (13)	20 (6.9)	0.04

Allergic	0 (0)	1 (0.2)	0.71	2 (1.6)	2 (0.7)	0.34
Loss of smell	6 (3.6)	32 (7.9)	0.04	3 (2.4)	3 (1)	0.25
Loss of taste	11 (6.6)	29 (7.2)	0.48	2 (1.6)	2 (0.7)	0.34
Gastrointestinal symptom	2 (1.2)	6 (1.5)	0.57	1 (0.8)	4 (1.4)	0.53
Chest pain	1 (0.6)	4 (1)	0.55	1 (0.8)	3 (1)	0.66
Dizziness	66 (39.8)	139 (34.5)	0.44	24 (19.5)	32 (11)	0.51

[a] *P* value from chi-square test or Fisher's exact test.

Table S8. Distribution of the intervals for reinfection by type of vaccine.

Reinfection interval	No.	Median	IQR[a]	Mean (95% CI[b])	*P* value[c]
Total	712	164.50	129.00–419.00	265.78 (253.81–277.76)	
Types of vaccine					
Mix†	107	232.00	126.00–424.00	277.03 (65.20–398.80)	
mRNA alone‡	373	202.00	137.00–425.00	286.81 (111.62–292.38)	0.167
Vector alone§	30	262.00	119.00–410.00	270.40 (27.16–496.84)	

Data are presented as n or %.

[a] IQR, interquartile range.

[b] CI, confidence interval.

[c] Kaplan–Meier analyses were performed to determine the effect of time of the reinfection interval by sex, age, presence of symptoms, vaccination, and underlying morbidities. Patients had been residents of Seoul since 1 January 2020. All patients were reinfected with SARS-CoV-2.

†Received vaccine only of mRNA (BNT162b2 (tozinameran, Pfizer-BioNtech) or mRNA-1273 (lasomeran, Moderna)) or Vector (Jcovden (Ad26.COV2.S, previously COVID-19 Vaccine Janssen) or ChAdOx1-S [recombinant] COVID-19 vaccine (AstraZeneca, ChAdOx1-S)) vaccines before February 24, 2022.

‡Received vaccine only of mRNA (BNT162b2 (tozinameran, Pfizer-BioNtech) or mRNA-1273 (lasomeran, Moderna)) vaccines before February 24, 2022.

§Received vaccine only of Vector (Jcovden (Ad26.COV2.S, previously COVID-19 Vaccine Janssen) or ChAdOx1-S [recombinant] COVID-19 vaccine (AstraZeneca, ChAdOx1-S)) vaccines before February 24, 2022.
